# The values and limitations of mathematical modelling to COVID-19 in the world: a follow up report

**DOI:** 10.1080/22221751.2020.1843973

**Published:** 2020-11-14

**Authors:** Yuanji Tang, Sherry Tang, Shixia Wang

**Affiliations:** a Applied NanoFemto Technologies, LLC, Lowell, MA, USA; b Department of Pathology, Southern California Permanente Medical Group, Riverside, CA, USA; c Department of Medicine, University of Massachusetts Medical School, Worcester, MA, USA

**Keywords:** COVID-19, SARS-CoV-2, modelling, pandemic, epidemiology

## Abstract

We previously described a mathematical model to simulate the course of the COVID-19 pandemic and try to predict how this outbreak might evolve in the following two months when the pandemic cases will drop significantly. Our original paper prepared in March 2020 analyzed the outbreaks of COVID-19 in the US and its selected states to identify the rise, peak, and decrease of cases within a given geographic population, as well as a rough calculation of accumulated total cases in this population from the beginning to the end of June 2020. The current report will describe how well the later actual trend from March to June fit our model and prediction. Similar analyses are also conducted to include countries other than the US. From such a wide global data analysis, our results demonstrated that different US states and countries showed dramatically different patterns of pandemic trend. The values and limitations of our modelling are discussed.

Since our first publication describing a simple mathematical model in late March 2020 [1], the total global population infected by COVID-19 increased from 2.4 million to 40 million and the total number of deaths from 165,000 to over 1,100,000 [2]. And more significantly, there is no sign that the pandemic is slowing down. COVID-19 continues to spread to more developing countries and geographic areas such as Brazil [3–5], India [6–10] and South Africa [11–14].

Our mathematical model was developed to simulate the course of this pandemic and try to predict how this outbreak might evolve [1]. We hope that the simple approach will be useful to monitor and project the near future course of pandemic in a given local area or a country.

Our original published report used the mathematical model to track the outbreaks of COVID-19 in the US and its selected states to identify the rise, peak, and decrease of cases within a given geographic population, as well as a rough calculation of accumulated total cases in this population from the beginning in March to the end of June 2020 [1]. It is everyone’s hope that the pandemic will slow down by that time.

The current report will review how well the later actual trend from March to June fit our previous model and prediction. Similar analyses are also conducted to include countries other than the US. From such a wide global data analysis, our model demonstrated the dramatically different pandemic patterns among different countries which will further stimulate more questions.

## Materials and methods

As previously described, our modelling analysis is done with the following steps:

First, the daily increase in cases (ΔN) was collected and used to calculate the daily growth rate (ΔN/N), which is the daily increase in cases (ΔN) divided by the total number of cases from the previous day (N). The daily growth rate curve is plotted.

Second, the 5-day moving average of the daily growth rate was calculated and plotted to minimize the potential measurement error and to smooth the curve of daily growth rate.

Third, 7–10 consecutive daily points on the smoothed curve were collected after the daily growth rate started going down but the daily new cases continue to rise.

Fourth, we fit the smoothed curve with exponential function based on the above 7- to 10-day data trend to get a decay factor (each region would have their individual decay factor).

Fifth, we assume that the decrease of the daily growth rate obeys exponential decay.

N(t)∼N(t0)exp⁡(t)

Finally, the decay factor was used to calculate and extend new daily growth rate, and to predict the future number of daily new cases. The future daily new case data was then used to calculate and extend the total cumulative cases as the future prediction.

## Results

### North America

**Figure 1.**
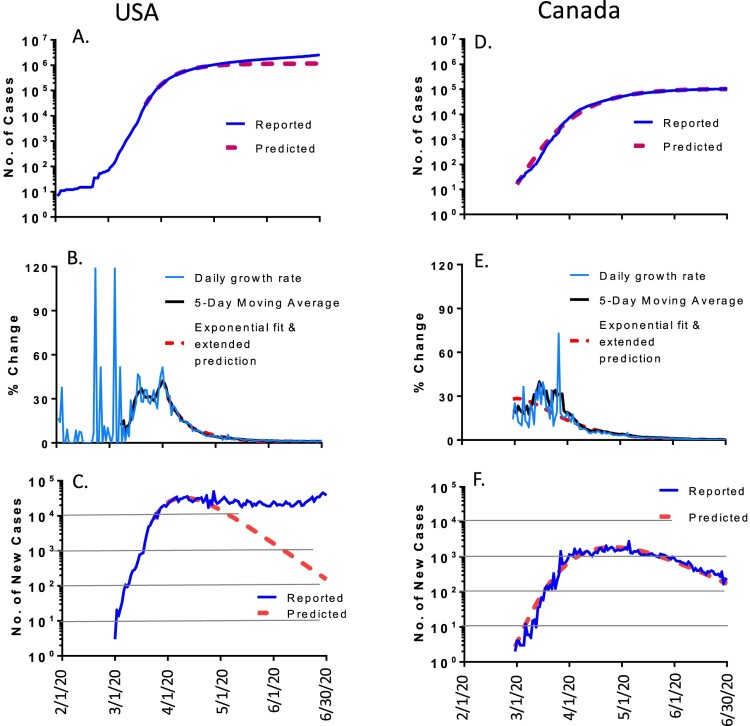
The COVID-19 pandemic trends in North America: USA (left panel) and Canada (right panel), by the end of June 2020. Total of cumulated COVID-19 cases in USA (A) and Canada (D): reported cases (blue) and predicted cases (red). Daily growth rate of COVID-19 cases in USA (B) and Canada (E): actual daily growth rate (blue), 5-day moving average of the growth rate (black) and exponential fix and predicted growth rate (red). Daily new COVID-19 cases in USA (C) and Canada (F): reported numbers (blue curve) and predicted numbers (red).

Although both are North America countries, USA and Canada had different trends in the COVID-19 pandemic (Figure 1). For the USA, the daily new cases first followed the prediction until close to the end of March 2020 when numbers took a major shift away from the predicted curve which should show a steady decrease of new cases (Figure 1(C)). However, the daily number of new cases for the entire country stayed at high levels and even went up by the end of June.

Matching with this pattern, the total number of accumulated cases also started to shift upward from predicted levels and ended with a much larger infected population by the end of June 2020: a total of 2.59 million people were infected, which is over 2 times higher than the predicted 1.2 million based on our modelling analysis (Figure 1(A)).

In contrast, the neighbouring country Canada presented a completely different pattern. The daily new cases (Figure 1(F)) and the total accumulated cases (Figure 1(D)), followed the prediction very well. By June 30, the reported accumulated cases in Canada were 0.103 million which is very close to the predicted 0.102 million (Table 1).

**Table 1.** The predicted and actual total numbers of COVID19 cases in US states and other countries included in the current report.

Prediction accuracy	Country or USA state	Predicted cases by 30 June 2020	Actual cases by 30 June 2020	Variations (%)
< 10% variation	New Zealand	1162	1169	0.6
	Canada	102,481	103,239	0.7
	United Kingdom	280,173	283,095	1.0
	Italy	227,408	240,310	5.6
	Spain	233,972	248,970	6.4
	France	151,081	162,936	7.8
	Germany	179,324	193,761	8.1
	Illinois, USA	136,752	143,184	4.7
	New Jersey, USA	162,070	171,637	5.9
Between 10%–50% variation	Australia	6971	7767	11.4
	S Korea	10,998	12,757	15.9
	Russia	512,833	634,437	23.7
	Pennsylvania, USA	67,234	91,138	35.5
	Massachusetts, USA	79,842	108,882	36.4
	New York, USA	291,982	398,504	36.5
> 50% variation	USA	1,152,297	2,590,552	124.8
	Brazil	179,324	1,864,681	939.8
	Michigan, USA	44,474	70,725	59.0
	Nevada, USA	7,816	18,591	137.9
	California, USA	47,403	232,274	389.9

### US states matching the modelling prediction (<10% variation of total cases by 30 June 2020)

**Figure 2.**
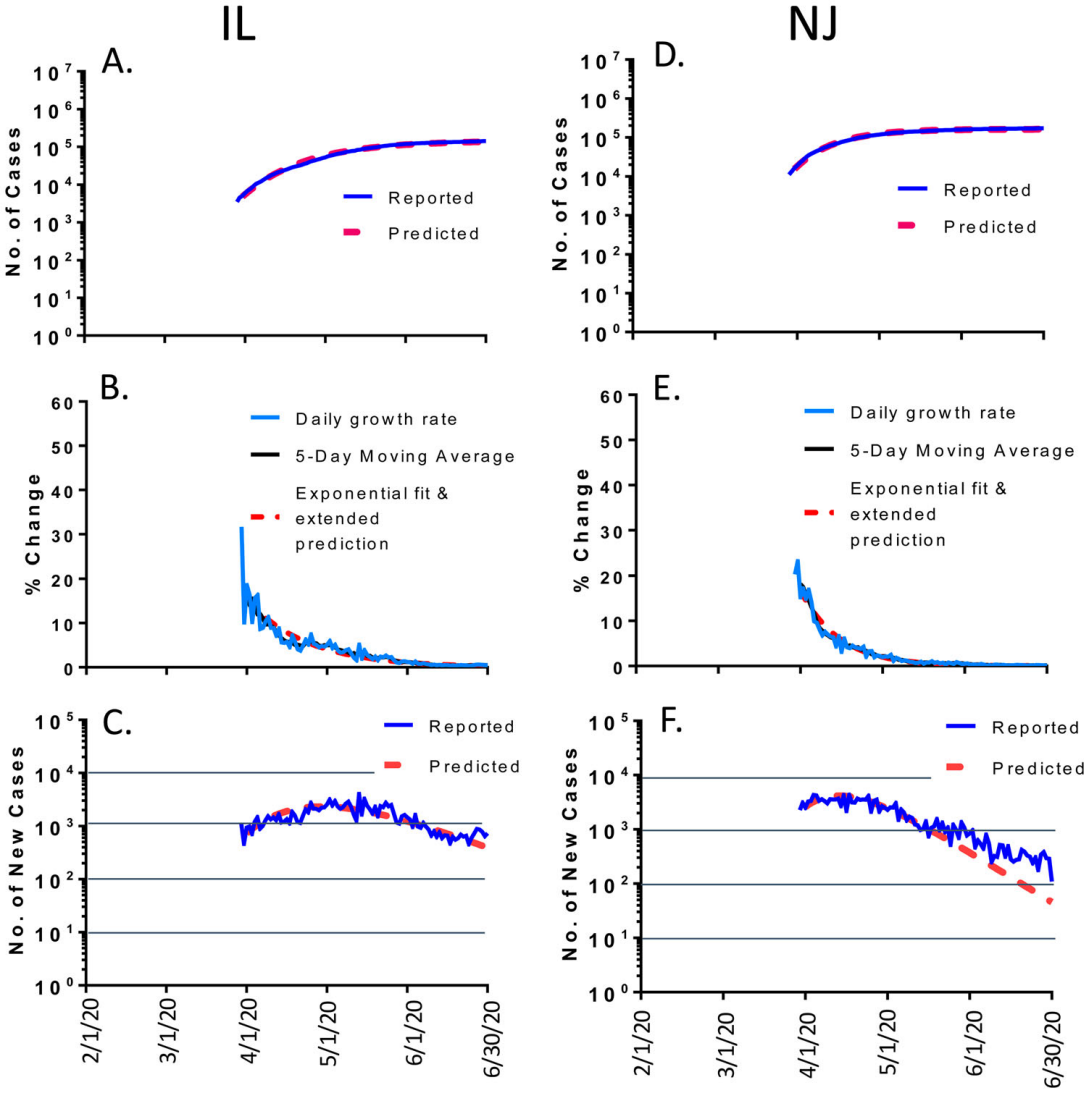
The COVID-19 pandemic trends in representative states in the USA, matching the modeling well (<10% variation of total cases by 30 June 2020): Illinois (IL, left panel) and New Jersey (NJ, right panel). Total cumulative COVID-19 cases in IL (A) and NJ (D): reported cases (blue) and predicted cases (red). Daily growth rate of COVID-19 cases in IL (B) and NJ (E): actual daily growth rate (blue), 5-day moving average of the growth rate (black) and exponential fix and predicted growth rate (red). Daily new COVID-19 cases in IL (C) and NJ (F): reported numbers (blue) and predicted numbers (red).

The same variability in outcome prediction was also observed among different states within the US. For example, the daily cases and the total accumulated cases in the states of Illinois (IL) and New Jersey (NJ) followed the prediction quite well (Figure 2). After the reported numbers of new cases reached the peak levels in early May in IL and middle April in NJ, the daily new cases gradually dropped steadily according to the math modelling prediction. The only exception is that the reduction of daily new cases in NJ became somewhat slower in June (Figure 2(F)). But the overall prediction was quite accurate. By June 30, 2020, the reported accumulated cases were 0.143 million in IL with 5% more cases than the predicted number of 0.136 million; and the reported accumulated cases were 0.172 million in NJ with 6% more than the predicted number of 0.162 million (Table 1).

### US states partially matching the modelling prediction (10–50% variation of total cases by 30 June 2020)

**Figure 3.**
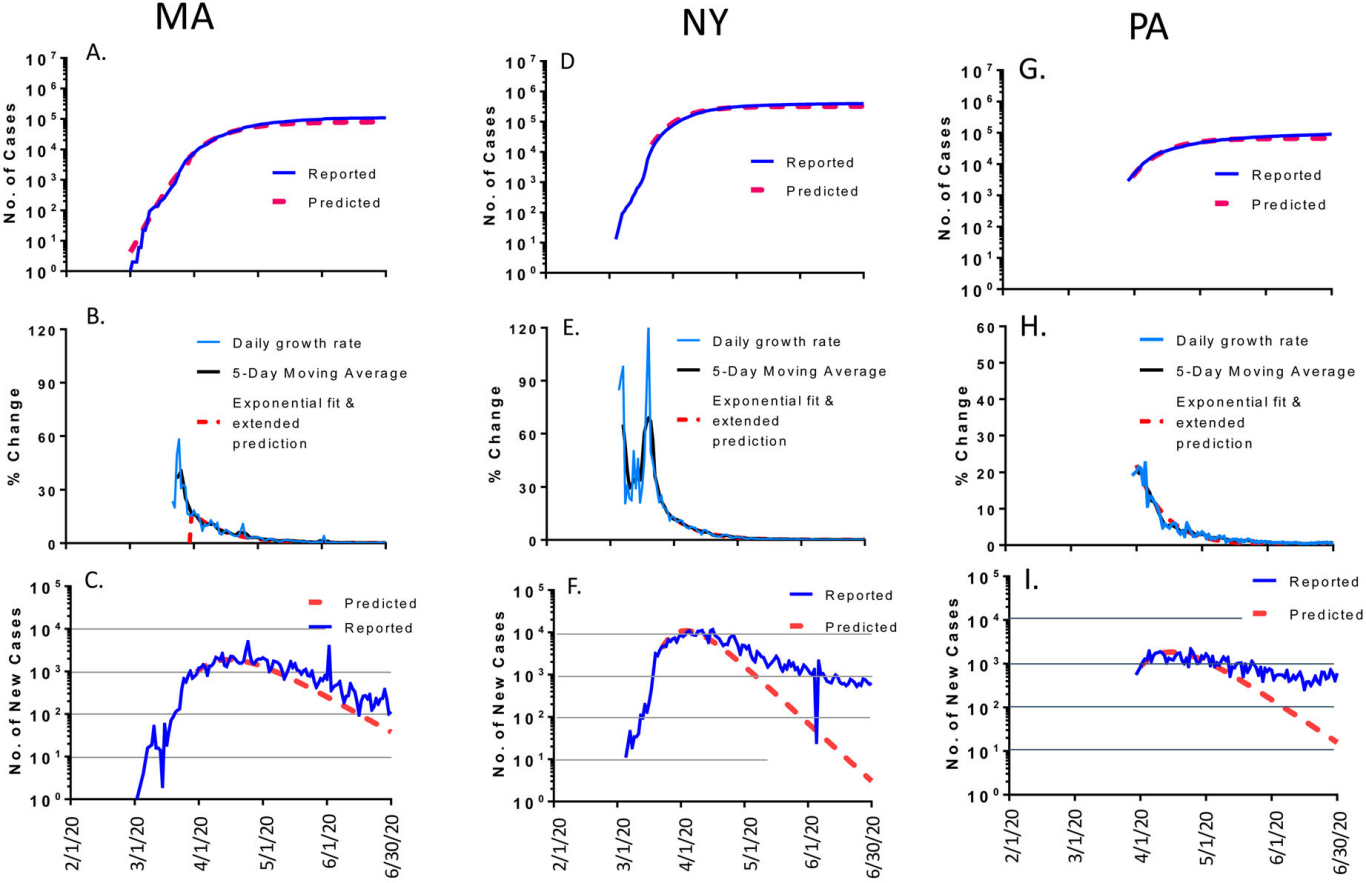
The COVID-19 pandemic trends in representative states in the USA, partially matching the modeling (10-50% variation of total cases by 30 June 2020): Massachusetts (MA, left panel), New York (NY, middle panel) and Pennsylvania (PA, right panel). Total cumulative COVID-19 cases in MA (A), NY (D) and PA (G): reported cases (blue) and predicted cases (red). Daily growth rate of COVID-19 cases in MA (B), NY (E) and PA (H): actual daily growth rate (blue), 5-day moving average of the growth rate (black) and exponential fix and predicted growth rate (red). Daily new COVID-19 cases in in MA (C), NY (F) and PA (I): reported numbers (blue) and predicted numbers (red).

Several US states such as Massachusetts (MA), New York (NY) and Pennsylvania (PA) showed a different pattern. The daily new cases and the reported accumulated cases, only partially aligned with the math modelling curves with clear divergence at the later time during the study period (Figure 3). After the daily numbers of new cases reached the peak levels in April in these three states, the number of daily new cases started to drop but the rates of drop became slower than what were predicted for these states. NY had the slowest drop, remaining about 1000 new cases per day by the end of June while it was predicted to be around 10 new cases per day. By June 30, 2020, the total number of accumulated cases was 0.109 million in MA which is 36% more than the predicted number of 0.08 million. At that time, the total number of accumulated cases was 0.399 million in NY, and 0.091 million in PA, also 36% more than predicted 0.292 million in NY and 0.067 million in PA, respectively (Table 1).

### US states poorly matching the modelling prediction (>50% variation of total cases by 30 June 2020)

**Figure 4.**
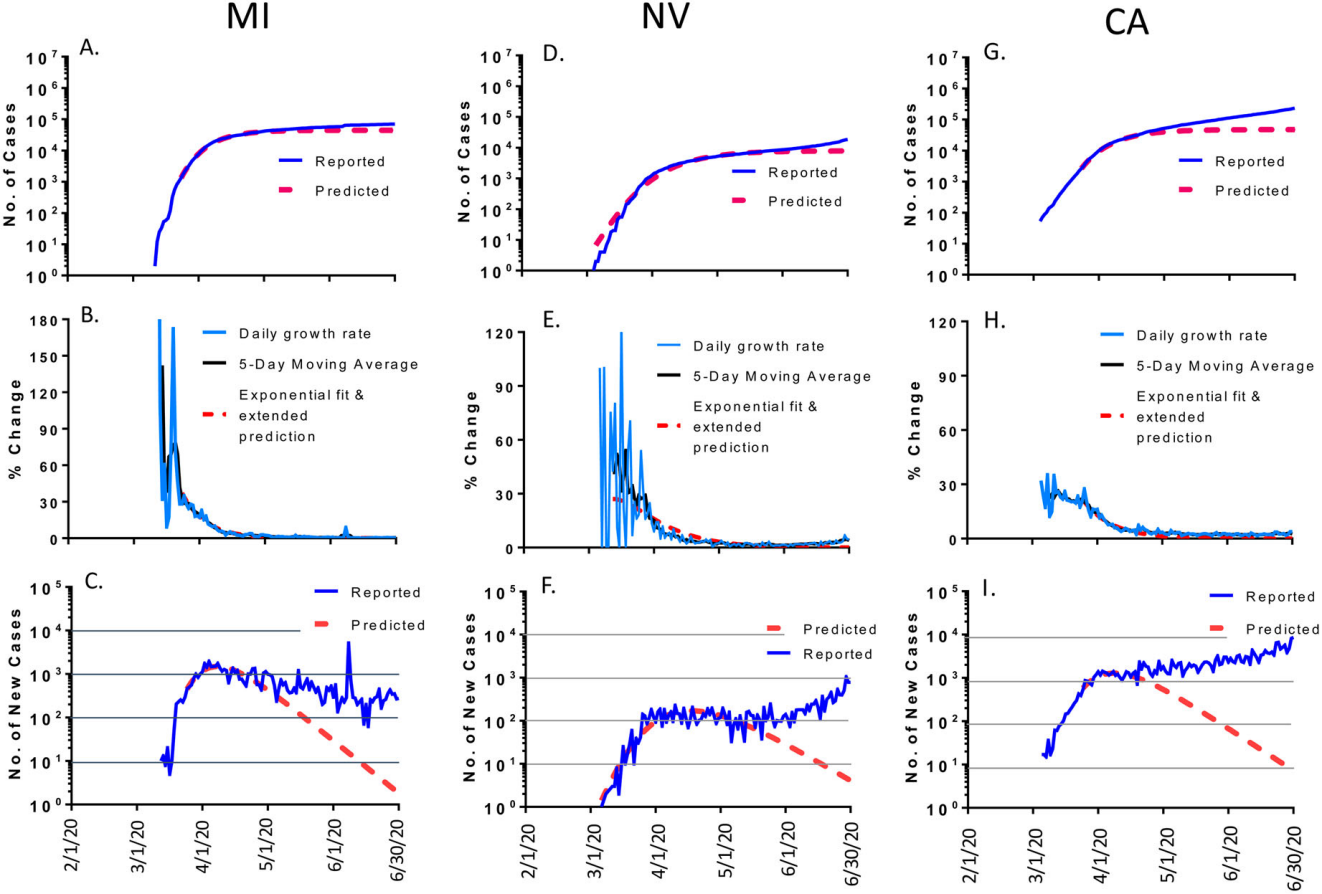
The COVID-19 pandemic trends in representative states in the USA, matching the modeling poorly (>50% variation of total cases by 30 June 2020): Michigan (MI, left panel), Nevada (NV, middle panel) and California (CA, right panel). Total cumulative COVID-19 cases in MI (A), NV (D) and CA (G): reported cases (blue) and predicted cases (red). Daily growth rate of COVID-19 cases in MI (B), NV (E) and CA (H): actual daily growth rate (blue), 5-day moving average of the growth rate (black) and exponential fix and predicted growth rate (red). Daily new COVID-19 cases in MI (C), NV (F) and CA (I): reported numbers (blue) and predicted numbers (red).

Several US states such as Michigan (MI), Nevada (NV) and California (CA) clearly showed significant divergence from what were predicted (Figure 4). In MI, after the number of daily new cases reached the assumed peak levels in April 2020 as predicted, that number did not drop significantly and stayed constant from April to June 2020 (Figure 4(C)). As a result, by June 30, 2020, the total number of accumulated cases was 0.071 million in MI, which is 1.59 times more than the predicted 0.044 million (Figure 4(A)). In NV and CA, new reported cases not only did not decline after the predicted peak but kept increasing from April to June 2020. By the end of June, the total number of accumulated cases was 0.019 million in NV, 2.36 times more cases than the predicted 0.0078 million; and the total number of accumulated cases was 0.232 million in CA, 4.9 times more cases than the predicted cases of 0.047 million (Table 1).

### Selected European countries

**Figure 5.**
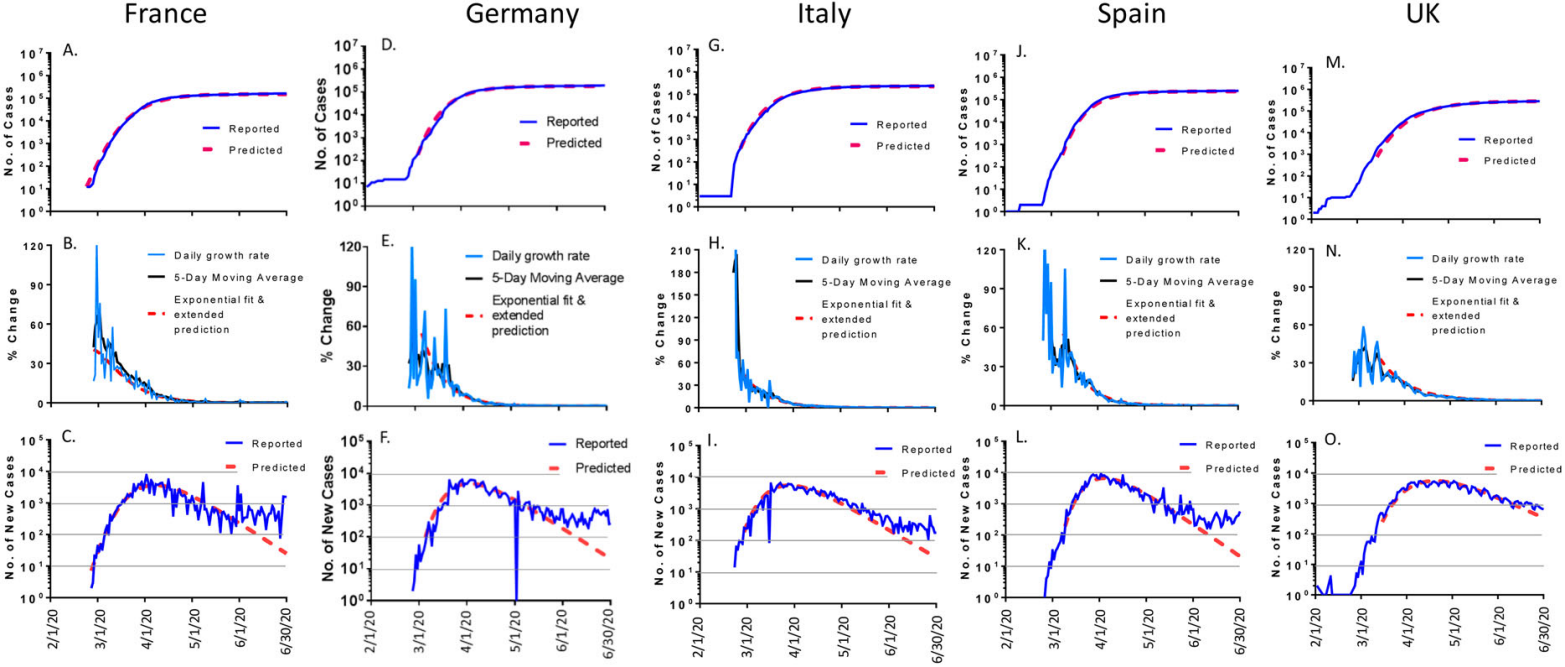
The COVID-19 pandemic trends in selected European countries by the end of June 2020: from left to right panels are France, Germany, Italy, Spain and British (UK). Total cumulative COVID-19 cases in France (A), Germany (D), Italy (G), Spain (J) and UK (M): reported cases (blue) and predicted cases (red). Daily growth rate of COVID-19 cases in France (B), Germany (E), Italy (H), Spain (K) and UK (N): actual daily growth rate (blue), 5-day moving average of the growth rate (black) and exponential fix and predicted growth rate (red). Daily new COVID-19 cases in France (C), Germany (E), Italy (I), Spain (L) and UK (O): reported numbers (blue) and predicted numbers (red).

Five European countries (France, Germany, Italy, Spain and United Kingdom) were included in our math modelling analysis for their COVID-19 epidemic trends (Figure 5). Although each of the countries had different numbers of COVID-19 cases, the epidemic trends were amazingly similar. The daily new cases and the total number of accumulated cases in these five countries aligned very nicely with the curves predicted by the math model. The daily new cases for all 5 countries gradually dropped after reaching the peak in March for Italy and in April for France, Germany, Spain and UK. The rate of drop in UK fit best to the predicted model (Figure 5(O)), while the drop in the other four countries started to deviate from the predicted model by early June (Figure 5(C, F, I, L)). By June 30, 2020, the total number of accumulated cases was 0.163 million in France, 7.9% more than the predicted 0.151 million; 0.194 million in Germany, 8.4% more than predicted 0.179 million; 0.24 million in Italy, 5.7% more than the predicted 0.227 million; 0.249 million in Spain, 6.4% more than the predicted 0.234 million; and 0.283 million in UK, 1.1% more than the predicted 0.28 million (Table 1).

### Oceania countries

**Figure 6.**
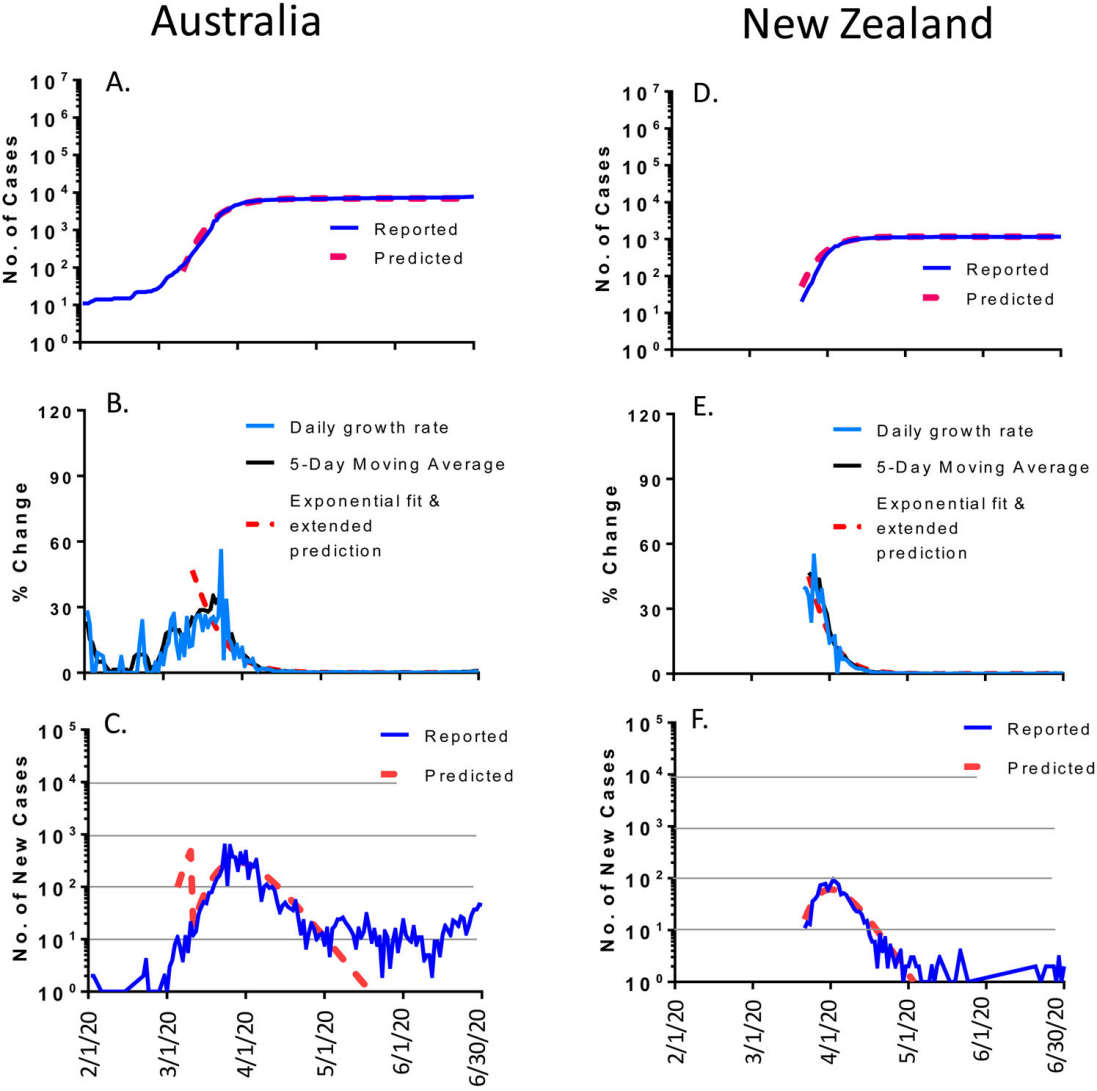
The COVID-19 pandemic trends in Oceania by the end of June 2020: Australia (left panel) and New Zealand (right panel). Total cumulative COVID-19 cases in Australia (A) and New Zealand (D): reported cases (blue) and predicted cases (red). Daily growth rate of COVID-19 cases in Australia (B) and New Zealand (E): actual daily growth rate (blue), 5-day moving average of the growth rate (black) and exponential fix and predicted growth rate (red). Daily new COVID-19 cases in Australia (C) and New Zealand (F): reported numbers (blue) and predicted numbers (red).

The COVID-19 pandemic showed another pattern in Australia and New Zealand (Figure 6). These two Oceania countries in the southern hemisphere are separated from the rest of the world and only saw small numbers of cases. The epidemic trends in both countries fit quite well to the math modelling’s predictions. The daily new cases gradually decreased after reaching the peak in late March for Australia and followed the predicted curve up to early May. Then, the daily new cases were kept at ∼10 cases per daily without further drop from early May to the end of June (Figure 6(C)). By June 30, 2020, the total accumulated cases in Australia were 7,767, 10% more than the predicted 6,971 million. While the scale of the COVID-19 pandemic is much smaller in New Zealand than many other countries, the trend followed nicely with the predicted pattern. The daily new cases gradually dropped after reaching the peak in early April and then followed very closely to the predicted curve (Figure 6(F)). The total number of accumulated cases in New Zealand was 1169, very similar to modelling predicted 1162 (Table 1).

### Countries from other regions

We also analyzed the COVID-19 epidemic trends in South Korea and Brazil, which showed two dramatically different patterns.

**Figure 7.**
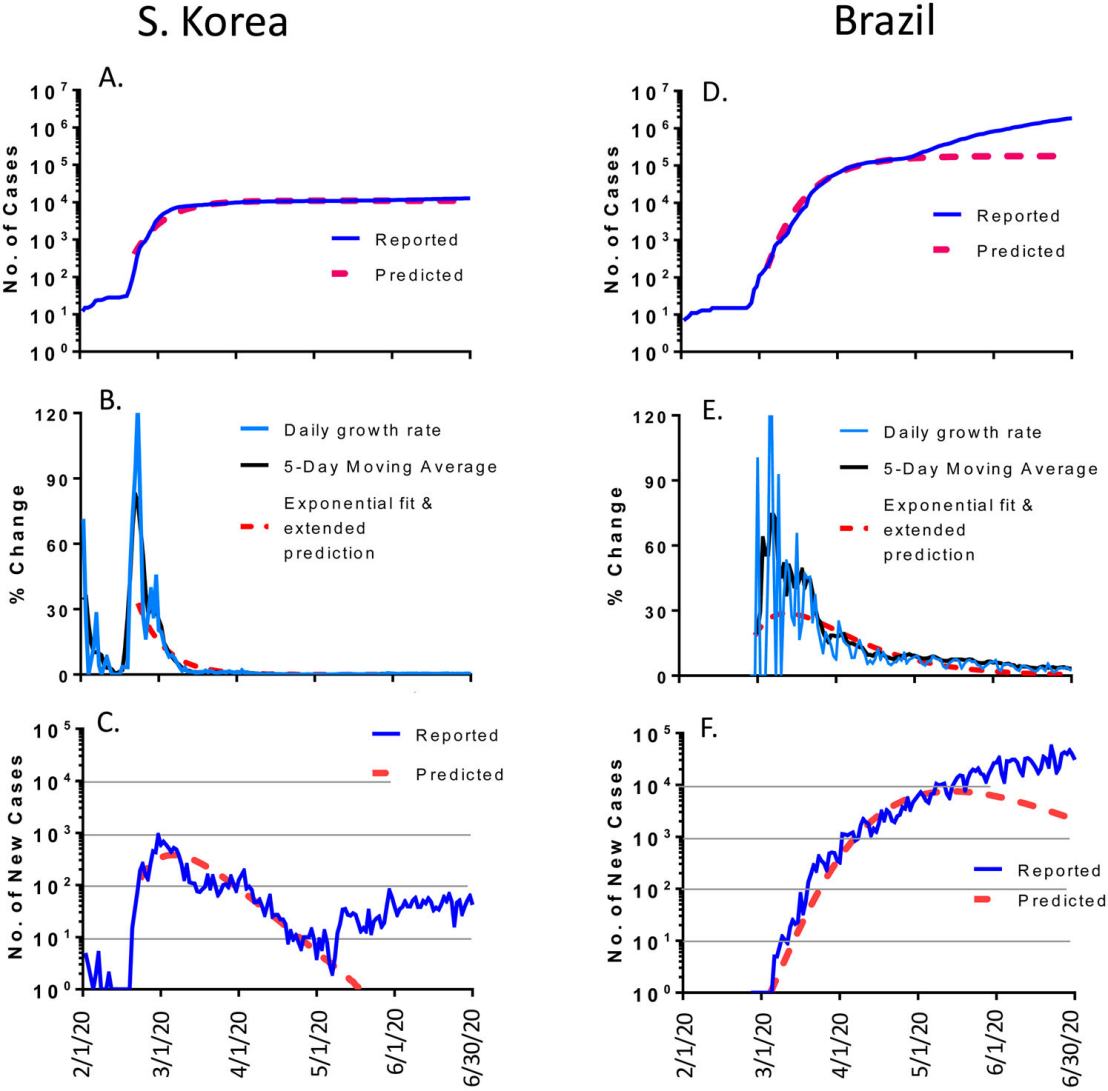
The COVID-19 pandemic trends in other regions by the end of June 2020: South Korea (left panel) and Brazil (right panel). Total cumulative COVID-19 cases in South Korea (A) and Brazil (D): reported cases (blue) and predicted cases (red). Daily growth rate of COVID-19 cases in South Korea (B) and Brazil (E): actual daily growth rate (blue), 5-day moving average of the growth rate (black) and exponential fix and predicted growth rate (red). Daily new COVID-19 cases in South Korea (C) and Brazil (F): reported numbers (blue) and predicted numbers (red).

South Korea was among the earliest countries reporting an outbreak of COVID-19 in February 2020. The peak of daily new cases was soon reached in early March 2020 and then gradually dropped and aligned with the predicted curve very well from early March to early May with less than 10 new cases per day (Figure 7(C)). However, more new daily cases appeared and kept rising from middle May to the end of June which diverged from the predicted curve. By 30 June 2020, the total number of accumulated cases in South Korea was 12,757, which is 16% more than the predicted number of 10,998 (Table 1).

The COVID-19 pandemic in Brazil showed a completely different pattern. It was predicted that the peak of daily new cases would be in early May 2020 and then the number should decline based on the math modelling. However, the daily new cases kept increasing at least to the end of June 2020, so that the daily new cases and total number of accumulated cases diverged significantly from the predicted curves. By 30 June 2020, the total number of accumulated cases in Brazil was 1.865 million, a stunning 10.4 times more cases than the predicted 0.179 million (Table 1).

## Discussion

This is a follow up analysis from the first report of using our mathematical model to project the COVID-19 pandemic in the US at both the country and state levels [1]. When the analyses were first done in March 2020, we set the end point of our prediction to the end of June 2020 because our math modelling predicted that COVID-19 cases will drop to very low level by that time. Since then, we have also used the same approach to analyze COVID-19 trend in other countries [3] including those reported in the current report.

Overall, we consider our mathematical modelling technically a success using the case data during the first part of the pandemic in a given geographic area. We were able to predict the peak level (or the turning point) of the regional trend. At least in the first period of case dropping, the prediction was generally accurate (except Brazil). For all five European countries, Canada, two Oceanic countries, and several US states, the decrease of daily new cases followed nicely with the predicted rate. In these countries and states, the total numbers of accumulated cases at the end of June were very close to predicted ones since the total number of accumulated cases is calculated based on the dynamics of daily new cases. This observation confirmed that the COVID-19 pandemic itself follows a common dynamic process which is governed mainly by the virology of this emerging SARS-CoV-2 virus including its rates of replication and transmission. The immediate interaction between the virus and the surrounding human population in the local region also plays important roles including social distancing, face mask and a standard 14-day quarantine following a suspected exposure with or without a local lock down. Most of these factors are reflected in our model development and the original prediction.

Most countries and states in the current analysis, although some reluctantly, took the same immediate responses as described above so a common pattern of peak point and subsequent decrease of new cases was observed. The unusual case in Brazil strongly indicated another scenario in which not even the basic immediate responses were taken, which allowed the virus to continue infecting large numbers of people who otherwise would be protected. In this case, the natural viral dynamic is disrupted, and no math model will be effective in predicting the next phase of transmission.

The more common deviation of actual viral trend from the predicted model is at the end of the decreasing phase when more new cases are re-emerging even in those areas where the viral dynamics first followed the prediction nicely. The causes for this finding are not clear and may be multifactorial. The most likely culprit would be the re-opening of society prematurely. Stores, restaurants, bars, gyms, and possibly schools re-opened. Next, the impact of more travelling should be considered. For example, why did many Southern states have more new emerging cases than Northern states in the summer of 2020 while the initial wave of COVID-19 was mainly in major cities like New York? It was suspected that the travellers from Europe may have contributed to the outbreaks in New York and Boston but it is not clear whether any cases were imported to Southern US states from South America when countries like Brazil had major outbreaks.

A truly useful model tool will need to incorporate more dynamic data including the changes of local public health policy, change between lockdown and re-opening of local daily activities, and the flow of travellers. However, the data shortage made such comprehensive effort very challenging. It was never our intention to develop a perfect model which will consider all possible variables. Rather, our modelling analysis is designed to provide simple and easy to understand information to the public and to provide basic prediction for the local government and public health agencies so they can take appropriate measures to respond to anticipated changes. Data from our analysis also provided a useful record for people living in those regions that the viral dynamics did not follow prediction. They can start further investigation to identify what particular factors at a given time period may have contributed to such deviations. With the useful control of other countries/states where the prediction was quite accurate, people can learn what needs to be done to maintain good control of COVID-19. This is highly important as the pandemic is still continuing, we need to learn what is effective in controlling the resurgence of this pandemic. Our analysis and prediction ended at the end of June, but the lessons learned from this period, especially the useful actions taken by regions different in our outcome analysis, would be critical to face the next wave of COVID-19 which is unfortunately re-emerging in multiple parts of the world at this time.
